# Profil épidémiologique et clinique des éviscérations oculaires au nord de l'Algérie, à propos de 136 cas

**DOI:** 10.48327/mtsi.v4i1.2024.383

**Published:** 2024-03-26

**Authors:** Amine HAMMA, Aïcha LAKHDAR FOUATIH, Lamine HAMMAD, Aïcha IDDER

**Affiliations:** 1 Service d'ophtalmologie du centre ho spitalo-universitaire de Béjaïa, Algérie; 2 Université de Béjaïa, Laboratoire de génétique médicale appliquée à l'ophtalmologie (LGMO), Algérie; 3 Établissement public hospitalier Souk-el Tenine, Béjaïa, Algérie; 4 Cabinet privé Les Eucalyptus, Alger, Algérie; 5 Université d'Oran Es-Senia, Laboratoire de génétique médicale appliquée à l'ophtalmologie (LGMO), N2A, Es Senia, Ouahran, Algérie

**Keywords:** Éviscération, Chirurgie mutilante, Épidémiologie, Oculoplastie, Endophtalmie, Traumatisme oculaire, Glaucome néovasculaire, Perforation cornéenne, Hôpital, Tlemcen, Oran, Béjaïa, Algérie, Maghreb, Afrique du Nord, Evisceration, Mutilating surgery, Epidemiology, Oculoplasty, Endophthalmitis, Ocular trauma, Neovascular glaucoma, Corneal perforation, Hospital, Tlemcen, Oran, Bejaia, Algeria, Maghreb, Northern Africa

## Abstract

**Introduction:**

La réalisation des chirurgies mutilantes en ophtalmologie est une décision lourde à prendre malgré des indications bien codifiées du fait des préjudices esthétique et moral que vont subir les patients. Cette chirurgie ne doit être envisagée qu'en dernier recours face à un œil non fonctionnel, douloureux et inesthétique, voire même en cas d'atteinte carcinologique après avoir épuisé toutes les alternatives conservatrices.

**Matériels et méthodes:**

Nous avons mené une étude observationnelle, rétrospective et exhaustive des dossiers médicaux des archives des services d'ophtalmologie du Centre hospitalier universitaire Dr. Tidjani Damardji de Tlemcen, de l’Établissement hospitalier spécialisé en ophtalmologie (EHS) d'Oran Hamou Boutlelis, de l’Établissement hospitalier spécialisé en ophtalmologie d'Oran Front de mer et du Centre hospitalier universitaire de Béjaïa (unité Franz Fanon), dans le but de préciser le profil épidémio-clinique des patients ayant bénéficié d'une éviscération oculaire au nord de l'Algérie durant la période du 1er janvier 2008 au 31 décembre 2014.

**Résultats et discussion:**

Nous avons recensé 136 patients, ce qui représente un taux d'admission de 0,13 % dans l'ensemble de ces services. Nous avons noté une prédominance masculine avec un sex-ratio estimé à 1,4. L’éviscération a été réalisée principalement dans les suites d'un traumatisme oculaire dans 39 % des cas. La technique chirurgicale réalisée chez tous les patients était une éviscération classique non conservatrice dite des « quatre quadrants » ou des « quatre carrés » et ce sous anesthésie générale dans 55,9 % des cas. Des complications post-opératoires ont été retrouvées chez 19,8 % des patients de notre série. L'accessibilité aux ocularistes ainsi que la qualité des équipements prothétiques ont également été étudiées. L'ensemble des données recueillies a été comparé avec les données de la littérature médicale internationale.

**Conclusion:**

La prévention des chirurgies mutilantes passe par le diagnostic précoce et le traitement adapté des pathologies ophtalmologiques et des traumatismes. Perdre un œil est toujours vécu comme une tragédie et peut être dévastateur à tout âge, en retentissant sur l'image et l'estime de soi. Le soutien psychologique est donc indispensable.

## Abstract (en)

**Introduction:**

Despite the existence of well codified indications, the performance of mutilating surgery in ophthalmology is not an easy decision to take due to the aesthetic and moral damages that patients may suffer. This surgery should be considered as a last resort in the case of a non-functional, painful and unsightly eye or in the presence of an oncological involvement and after all conservative alternatives have been exhausted. This study aims to define the factors making it possible to favor ocular evisceration, which is the least mutilating of the above-mentioned surgeries, by determining the epidemiological and clinical aspects of the patients. In addition, the results of the study will serve as a starting point for epidemiological surveillance and will guide preventive activities and the fight against blindness.

**Material & method:**

We conducted an exhaustive retrospective study of medical records from the archives of the ophthalmology departments of the Dr. Tidjani Damardji University Hospital Center in Tlemcen, the Specialized Hospital Establishment in ophthalmology of Oran Hamou Boutlelis, the Specialized Hospital Establishment in ophthalmology of Oran Front de mer and the University Hospital Center of Bejaia (unit Franz Fanon), in order to specify the epidemiological-clinical profile of patients who have undergone an ocular evisceration in the north of Algeria from January 1, 2008 to December 31, 2014.

**Results & discussion:**

We have identified 136 patients, representing an admission rate of 0,13% in all these services. We noted a slight male predominance with an estimated sex-ratio of 1.4. Evisceration was carried out mainly following an ocular trauma in 39% of cases. The surgical technique performed in all patients is a classic non-conservative evisceration of “four quadrants” or “four squares” under general anesthesia in 55.9% of cases. Post-operative complications were found in 19.8% of patients in our series, the main one being exteriorization of the intra-scleral implant in 9.5% of cases. This rate corresponds to the data in the literature, with figures between 0 and 67%. This complication may be in relation with the experience or even the competence of the surgeon. Accessibility to ocularists and the quality of prosthetic equipment were also studied. All the data collected were compared with data from the international medical literature. Our study carried out in the north of Algeria on ocular eviscerations, allowed us to deduce that this surgery is rarely carried out in ophthalmology. Its main indications are post-traumatic and post-infectious.

**Conclusion:**

The prevention of mutilating surgeries requires early diagnosis and appropriate treatment of ophthalmological pathologies and trauma. Losing an eye is always experienced as a tragedy and can be devastating at any age, affecting self-image and self-esteem. Psychological support is therefore essential.

## Introduction

La réalisation des chirurgies mutilantes en ophtalmologie est une décision bien lourde à prendre malgré des indications bien codifiées du fait des préjudices, esthétique et moral, que vont subir les patients [7]. Ces chirurgies comprennent : l’énucléation qui consiste à enlever le globe oculaire en totalité avec section du nerf optique le plus à distance possible du pôle postérieur du globe [10]; l’éviscération qui consiste à vider le contenu du globe oculaire en laissant la coque sclérale, la conjonctive et les muscles oculomoteurs en place. Ces deux techniques peuvent être réalisées pour les mêmes indications, mais l’énucléation devient obligatoire en cas de tumeur endo-oculaire. Elles sont suivies

d'une implantation intra-orbitaire qui comblera le vide créé et facilitera l'adaptation d'une prothèse oculaire à visée esthétique. L'exentération consiste en l'exérèse en bloc du contenu de l'orbite et est indiquée dans les cas d'envahissement orbitaire de différentes pathologies tumorales, en particulier d'origine palpébrale ou oculaire. L'appareillage se fait grâce à une épithèse [8, 12].

La chirurgie mutilante ne doit être envisagée qu'en dernier recours, après avoir épuisé toutes les autres alternatives thérapeutiques conservatrices du globe oculaire, à savoir : les kératoprothèses, indiquées en cas de cécité cornéenne bilatérale [9], les injections rétrobulbaires de xylocaïne 2 % (2 ml) et d'alcool 60 % (1 ml) [16], indiquées pour calmer les douleurs rebelles aux traitements (essentiellement celles dues au glaucome néovasculaire [22, 23]), les verres scléraux à visée esthétique qui sont indiqués beaucoup plus en cas de phtyse du globe [21]. Si leur mise en place est douloureuse, on doit réaliser préalablement un recouvrement conjonctival.

Cette étude vise à définir les facteurs permettant de privilégier l’éviscération oculaire qui est la moins mutilante des chirurgies, en déterminant les aspects épidémiologiques et cliniques des patients qui ont en bénéficié. De plus, les résultats de l’étude serviront de point de départ pour la surveillance épidémiologique et orienteront judicieusement les activités préventives et de lutte contre la cécité.

## Matériels et méthodes

**Figure 1:**
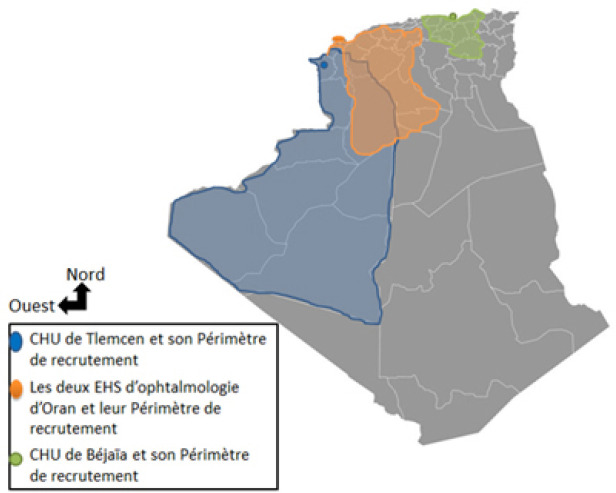
Carte de l'Algérie avec identification des périmètres de recrutement des différentes structures hospitalouniversitaires incluses dans l’étude Map of Algeria with identification of the recruitment perimeters of the different hospital-university structures included in the study

Nous avons mené une étude observationnelle rétrospective exhaustive des dossiers médicaux des patients ayant bénéficié d'une éviscération oculaire au nord de l'Algérie durant la période du 1^er^ janvier 2008 au 31 décembre 2014. Pour cela, 4 structures hospitalo-universitaires ont été choisies comme échantillon (Fig. 1) :

- Le service d'ophtalmologie du Centre hospitalier universitaire Dr. Tidjani Damardji de Tlemcen, situé à l'extrême nord-ouest de l'Algérie au chef-lieu de la wilaya de Tlemcen, avec un périmètre de recrutement des patients dépassant les 300 km couvrant la wilaya de Tlemcen et les wilayas limitrophes d'Aïn Témouchent, de Naâma, ainsi que les wilayas du sud de l'Algérie dépourvus d'hôpitaux universitaires telles que les wilayas d'Elbayadh, de Béchar et d'Adrar.
- L’établissement hospitalier spécialisé en ophtalmologie d'Oran Hamou Boutlelis.
- L’établissement hospitalier spécialisé en ophtalmologie d'Oran Front de mer, situés à Oran, qui représente la 2^e^ wilaya d'Algérie en termes de population.

S'agissant de centres de référence en ophtalmologie, ces deux établissements ont un recrutement national. Cependant, leur périmètre de recrutement avoisine les 250 km comprenant les wilayas de Mostaganem, de Mascara, de Relizane, d'Aïn Témouchent, de Chlef, de Saïda, de Tissemsilt et de Tiaret et le service d'ophtalmologie du Centre hospitalier universitaire de Béjaïa (unité Franz Fanon), situé au centre-ville de la wilaya de Béjaïa au centre de l'Algérie, avec un périmètre de recrutement des patients avoisinant les 180 km, comprenant les wilayas de Bouira, de Tizi Ouzou, de M'Sila, de Jijel, de Bordj Bou Arreridj, de Mila et de Sétif.

Nous avons exclu de notre étude les patients ayant bénéficié d'autres types de chirurgies mutilantes ophtalmologiques. Le recueil et l'analyse des données ont été réalisés grâce au logiciel Epi Info 7. Les données en question concernent l’âge, le sexe, les principales indications chirurgicales, les techniques et les matériaux utilisés, l'accessibilité aux ocularistes ainsi que la qualité des équipements prothétiques. L'exploitation de ces données nous a permis de réaliser des statistiques dont les résultats ont été exprimés sous forme de diagrammes et /ou tableaux de fréquences, puis comparés aux données de la littérature.

## Résultats

Durant 7 ans, 107 328 patients ont été hospitalisés au sein des services d'ophtalmologie des différentes structures hospitalo-universitaires concernées par notre étude. Parmi eux, 136 patients ont bénéficié d'une éviscération, à savoir 37 patients à Tlemcen, 72 patients à Oran (en regroupant les deux établissements hospitaliers spécialisés en ophtalmologie), 27 patients à Béjaïa. Ce qui représente un taux d'admission de 0,13 % pour cette chirurgie. Un total de 52 422 interventions chirurgicales a été réalisé dans ces structures durant la période étudiée. De ce fait, les 136 éviscérations représentent un pourcentage de 0,26 % de l'ensemble des actes opératoires pratiqués et 91,9 % de l'ensemble des chirurgies ophtalmologiques mutilantes faites durant la même période.

Le nombre moyen de patients éviscérés en réunissant les données des 4 structures étudiées était estimé à 19,5 cas/an, avec un minimum de 11 cas durant l'année 2010 et un maximum de 28 cas durant l'année 2014.

Durant l’évolution saisonnière des éviscérations, on observe une prédominance hivernale estimée à 53 cas; le taux le plus bas était relevé durant la saison estivale avec 17 cas.

La durée d'hospitalisation moyenne était de 6 jours incluant le jour précédant l'intervention et 5 jours postopératoires. Les extrêmes étaient de 1 jour pour un seul patient sorti contre avis médical et 18 jours pour des patients ayant nécessité une hospitalisation au préalable. Nous avons noté une légère prédominance masculine avec un sex-ratio estimé à 1,4.

Les âges extrêmes rencontrés dans notre série étaient de 10 ans à 97 ans, soit une étendue de 87 ans avec une moyenne d’âge de 58 ans et un intervalle de confiance à 95 % compris entre 56,1- 59,9 ans (Tableau I). Nous avons relevé un pic de fréquence entre 61 et 80 ans avec un total de 39 patients, soit près de 28,7 %. La médiane d’âge était de 57 ans et l'intervalle interquartiles entre 37 et 76 ans.

**Tableau I:** Répartition des patients selon les tranches d’âge au moment de l'intervention des 4 services d'ophtalmologie durant la période allant du 01/01/2008 au 31/12/2014 Distribution of patients according to age groups at the time of the intervention in the 4 ophthalmology departments during the period from 01/01/2008 to 31/12/2014

	EHS ORAN		CHU TLEMCEN		CHU BEJAIA		TOTAL	
Tranches d’âge	Nombre de cas	%	Nombre de cas	%	Nombre de cas	%	Nombre de cas	%
**0-20 ans**	4	6	2	5	3	11	9	6,6
**21-40 ans**	19	26	8	22	4	14	31	22,8
**41-60 ans**	20	28	12	32	4	14	36	26,5
**61-80 ans**	21	29	10	27	8	30	39	28,7
**81-100 ans**	8	11	5	14	8	30	21	15,4
**Total**	**72**	**100**	**37**	**100**	**27**	**100**	**136**	**100**

Toutes les éviscérations étaient monoculaires avec une prédominance sur les yeux gauches dans 55 % des cas.

**Figure 2:**
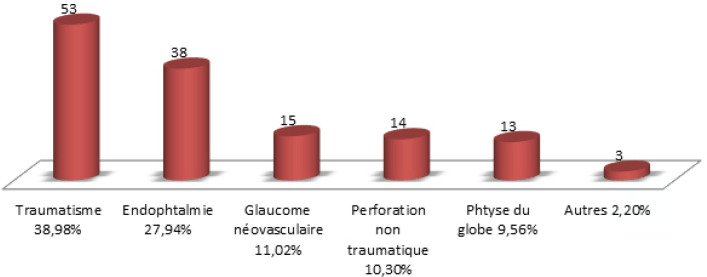
Indications d’éviscération des 4 services d'ophtalmologie durant la période allant du 01/01/2008 au 31/12/2014 Evisceration indications in the 4 ophthalmology departments during the period from 01/01/2008 to 31/12/2014

Concernant les données cliniques de la population étudiée, les antécédents de traumatisme oculaire étaient retrouvés dans 41 % des cas. Les principales indications d’éviscération retrouvées dans notre série étaient essentiellement des étiologies traumatiques dans 39 % des cas, suivies des endophtalmies qui représentaient 28 % puis vient le glaucome néovasculaire rebelle aux thérapeutiques usuelles avec 11 %, puis les perforations cornéennes avec 10 % et les phtyses du globe oculaire retrouvées dans 10 % des cas (Fig. 2).

D'un autre côté, les indications purement esthétiques chez des patients sans notion de douleur oculaire ne représentaient que 12 % des cas (Tableau II).

**Tableau II:** Détails des indications d’éviscération des 4 services d'ophtalmologie durant la période allant du 01/01/2008 au 31/12/2014 Details of evisceration indications in the 4 ophthalmology departments during the period from 01/01/2008 to 31/12/2014

Indications	Cadre étiologique	EHS Oran			CHU Tlemcen			CHU Béjaïa			
			Total	%		Total	%		Total	%	Total
Perforation	Maladies de la surface : dystrophie cornéenne, syndrome sec, kératolyse aseptique	9	28	39	2	4	10,8	1	1	3,7	24,3
	Infection oculaire	8			1			0			
	Traumatisme éclatement du globe	5			0			0			
	Ulcère sur trauma ancien	3			1			0			
	Brûlure chimique	3			0			0			
Phtyse, buphtalmie, glaucome absolu hyperalgique	Après chirurgie du décollement de rétine	12	25	35	0	14	37,8	0	15	55,6	39,7
	Post traumatique	9			14			11			
	Après chirurgie de la cataracte	2			0			0			
	Après kératoplastie transfixiante	1			0			0			
	Glaucome absolu	1			0			4			
Infection grave (abcès endophtalmie panophtalmie)	Après chirurgie de la cataracte	10	13	18	9	12	32,4	6	8	29,6	24,3
	Après chirurgie du décollement de rétine	2			0			0			
	Sur paralysie faciale	1			0			0			
	Après chirurgie du glaucome	0			1			2			
	Autres	0			2			0			
Esthétique	Atrophie du globe ou buphtalmie	6	6	8	7	7	18,9	3	3	11,1	11,8
**TOTAL**			**72**	**100**		**37**	**100**		**27**	**100**	**100**

Chez les patients âgés de moins de 40 ans, les traumatismes oculaires étaient les principales indications d’éviscération retrouvées, alors qu'au-delà de 40 ans, les infections oculaires primaient.

**Figure 3:**
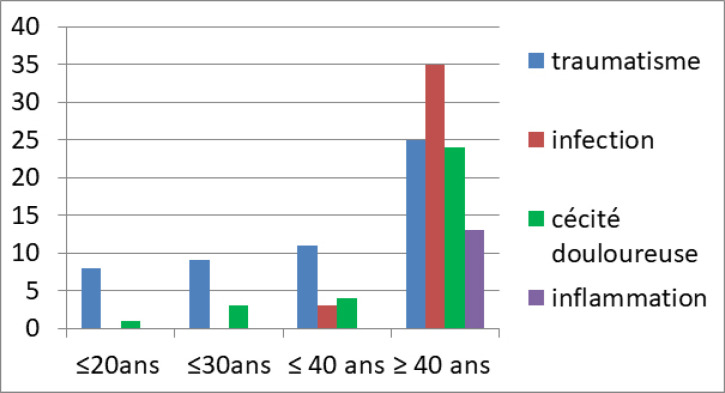
Indications d’éviscération selon l’âge des 4 services d'ophtalmologie durant la période allant du 01/01/2008 au 31/12/2014 Evisceration indications according to age in the 4 ophthalmology departments during the period from 01/01/2008 to 31/12/2014

**Figure 4:**
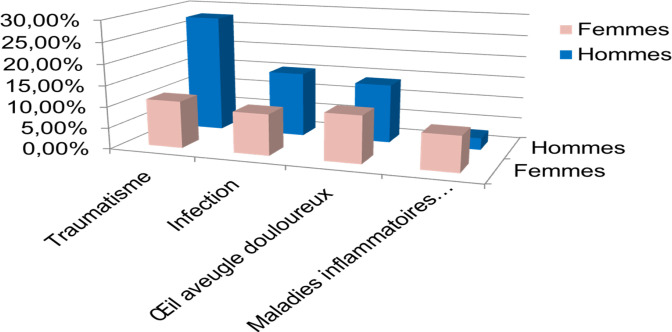
Indications d’éviscération selon le sexe des 4 services d'ophtalmologie durant la période allant du 01/01/2008 au 31/12/2014 Evisceration indications according to gender in the 4 ophthalmology departments during the period from 01/01/2008 to 31/12/2014

Concernant les étiologies inflammatoires et perforatives de la surface oculaire, elles n’étaient retrouvées qu'au-delà de l’âge de 40 ans (Fig. 3). Les étiologies traumatiques étaient plus fréquentes chez les hommes. A l'inverse, les étiologies inflammatoires perforatives étaient plus fréquemment retrouvées chez les femmes (Fig. 4).

**Figure 5:**
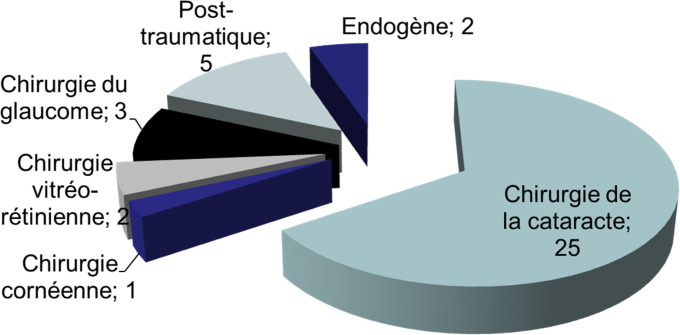
Répartition des endophtalmies retrouvées en fonction de leurs étiologies des 4 services d'ophtalmologie durant la période allant du 01/01/2008 au 31/12/2014 Distribution of endophathalmitis according to their etiologies in the 4 ophthalmology departments during the period from 01/01/2008 to 31/12/2014

Les endophtalmies occupaient la 2^e^ place en matière d'indication des éviscérations, retrouvées dans 27,9 % des cas. Elles se répartissent comme suit : 81,6 % d'endophtalmies post-opératoires et 18,4 % réparties entre endophtalmies post-traumatiques et endogènes (Fig. 5).

Concernant les techniques opératoires utilisées, l'anesthésie générale a été pratiquée chez 76 patients ce qui représente 55,9 % des cas, alors que les 60 patients restants ont bénéficié d'une anesthésie loco-régionale avec une sédation.

L'anesthésie loco-régionale a été réservée dans la plupart des cas aux patients à haut risque anesthésique (cardiopathie, âge très avancé…) sauf à l'EHS d'Oran où elle a été pratiquée de manière quasi-systématique.

**Figure 6:**
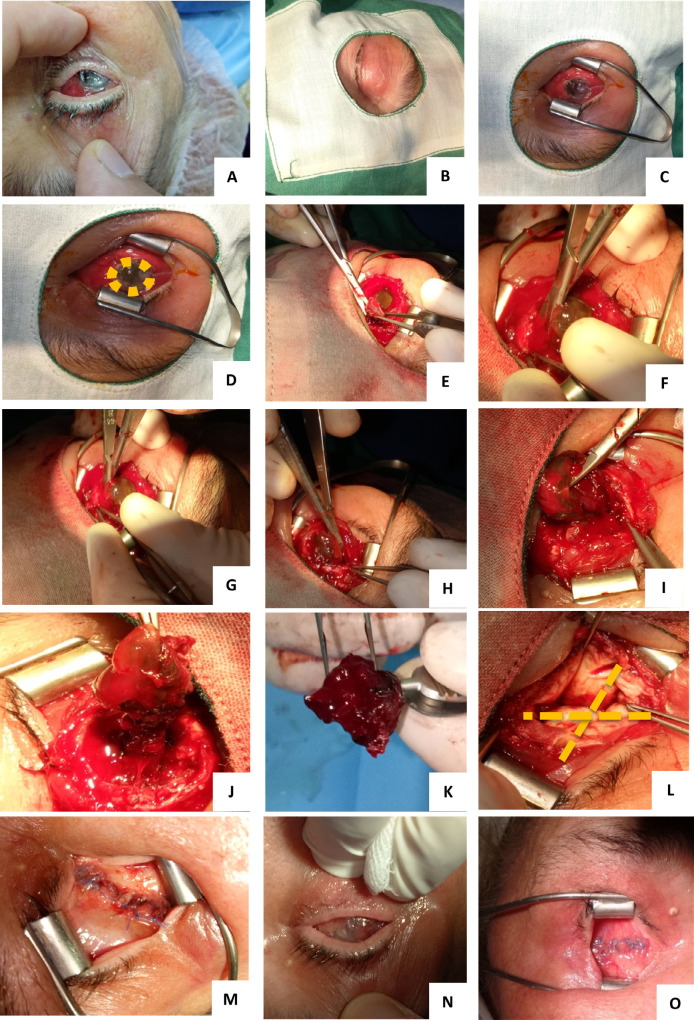
Les différentes étapes de la technique d’éviscération des « quatre quadrants » The different stages of the “four quadrants” evisceration technique

La technique chirurgicale réalisée chez tous les patients était une éviscération classique non conservatrice, technique des « quatre quadrants » ou des « quatre carrés » (Fig. 6). L'implantation intra-sclérale a été réalisée chez 78 patients (soit 57,4 % des cas), où 19 patients ont reçu un implant en polyméthacrylate de méthyle et 59 patients un implant en silicone. Le taux d'implantation a varié entre les différentes structures. Aux CHU de Béjaïa et de Tlemcen, les patients non implantés représentaient un pourcentage faible incluant essentiellement les cas d'endophtalmie non contrôlée, alors que pour l'EHS d'Oran plus de la moitié des patients éviscérés n’était pas implantée quelle que soit l’étiologie.

A. Œil présentant une endophtalmie chronique. B-C. Double désinfection cutanée et des culs de sac conjonctivaux. D. Péritomie : désinsertion conjonctivale sur 360° suivie de préférence par un amarrage des 4 muscles droits. E. Résection de la cornée par une incision au couteau de 15 puis aux ciseaux. F-G-H. Réalisation d'un plan de clivage entre la sclère et la supra-choroïde sur 360°. I-J. Éviscération du globe oculaire en bloc. K. Contenu du globe oculaire éviscéré. L. Aspect de la sclère après éviscération et réalisation de quatre traits de refend. M. Aspect post opératoire immédiat. N. Mise en place d'un conformateur. O. Aspect à j 7 post-opératoire

Les suites opératoires étaient les mêmes pour toutes les structures : la mise en place d'un conformateur jusqu’à ce qu'une prothèse oculaire soit confectionnée, la réalisation d'un pansement compressif pendant 24 heures, l'application d'une pommade associant un antibiotique et un corticoïde trois à quatre fois par jour pendant 10 jours, une antibiothérapie par voie générale maintenue pendant 7 jours, et une corticothérapie de courte durée. La prothèse personnalisée a été mise en place après 6 semaines en moyenne.

Dans notre série, 27 patients ont présenté des complications post opératoires, comprenant 11 cas de déhiscences conjonctivales, 3 cas d'infections du matériel d'implantation intra-scléral, et 13 cas d'extériorisations de ce matériel (Tableau III).

**Tableau III:** Complications post-opératoires retrouvées après éviscération dans les 4 services d'ophtalmologie durant la période allant du 01/01/2008 au 31/12/2014 Post-operative complications found after evisceration in the 4 ophthalmology departments during the period from 01/01/2008 to 31/12/2014

Complications	EHS ORAN	CHU TLEMCEN	CHU BEJAIA	TOTAL
**Infection de la bille**	2	0	1	3
**Déhiscence conjonctivale**	10	1	0	11
**Extériorisation de la bille**	9	2	2	13
**Total**	**21**	**3**	**3**	**27**

Le nombre d'ocularistes au nord de l'Algérie étant restreint, l'accès à ces derniers était difficile pour les patients contraints de traverser des distances supérieures à 200 km dans 22 % des cas (Tableau IV).

**Tableau IV:** Distances parcourues par les patients éviscérés pour accéder à un oculariste dans les 4 services d'ophtalmologie durant la période allant du 01/01/2008 au 31/12/2014 Distances traveled by eviscerated patients to reach an ocularist in the 4 ophthalmology departments during the period from 01/01/2008 to 31/12/2014

Distance parcourue	0-50 km	51-100 km	101-150 km	151-200 km	> 200 km
**Effectif**	45	22	19	16	28
**%**	34,6	16,9	14,6	12,3	21,5

La qualité des équipements prothétiques a été jugée sur 2 critères principaux qui sont :

- le type de prothèse utilisée, à savoir personnalisée et adaptée aux mesures du patient chez 25 % des patients, ou standard sur gabarit chez 71 % alors que 4,4 % des patients sont restés sans prothèse;
- la mobilité de la prothèse, bien que ce dernier critère dépende essentiellement de la technique opératoire utilisée. Nous avons noté 41 % de prothèses mobiles lors des mouvements de l’œil adelphe.

## Discussion

Au travers notre étude portant sur les patients éviscérés au nord de l'Algérie, nous avons pu atteindre nos objectifs en recueillant et en analysant divers paramètres concernant cette catégorie de patients, allant des indications opératoires jusqu’à la qualité de leurs équipements prothétiques, tout en précisant leur profil épidémiologique. En effet, ces données vont servir de repère pour connaître l’état des lieux en matière de chirurgies mutilantes et ce afin d'améliorer ultérieurement la qualité des soins qui leur sont proposés voire de prévenir le recours à ce genre de chirurgie.

Il s'agit de la première étude algérienne multicentrique portant sur ce sujet. Nous espérons apporter un plus à la littérature en identifiant les caractéristiques cliniques et épidémiologiques des éviscérations oculaires dans cette région. En effet, d'après nos recherches, aucune autre étude abordant ce thème n'a été publiée en Algérie jusqu’à présent.

Le caractère rétrospectif de notre étude limite nos résultats. Étant donné que l’éviscération oculaire est considérée comme un recours ultime face à une pathologie oculaire délabrante, cécitante et potentiellement douloureuse, c'est pour cela que nous n'avons pas pu préciser les alternatives thérapeutiques dont les patients ont bénéficié avant que cette chirurgie mutilante ne soit indiquée. Par ailleurs, le ressenti et l’état psychologique des patients n'ont pas pu être répertoriés au moment opportun. Les difficultés furent rencontrées essentiellement lors du recueil des données à partir des dossiers médicaux. En effet, 8 dossiers incomplets ont été retrouvés où certains diagnostics reportés sur les dossiers se confondaient avec les motifs d'hospitalisation. D'un autre côté, la non-collaboration des ocularistes locaux lors de l'enquête sur la qualité des équipements prothétiques était un obstacle majeur car nous aurions pu enrichir notre étude en détaillant les différents procédés de fabrication et les matériaux utilisés. La plupart des études auxquelles nous avons confronté nos résultats s'intéressaient aux chirurgies mutilantes en général et pas particulièrement aux éviscérations.

La fréquence de l'intervention dans notre série était estimée à 0,4 % de l'ensemble des actes opératoires. Ce taux est très largement inférieur aux taux retrouvés dans d'autres études présentées dans le tableau V.

**Tableau V:** Fréquence des chirurgies mutilantes par rapport à l'ensemble des actes opératoires au sein des services d'ophtalmologie Frequency of mutilating surgeries compared to all surgical procedures in ophthalmology departments

Pays		Fréquence	Type de chirurgie	Réf.
Togo		1,2 %	EVI +ENU +EXE	[25]
Népal		1,4 %	EVI +ENU	[19
Nigeria	Ebonyi	5 %	EVI +ENU +EXE	[18]
	Sokoto	0,76 %	EVI +ENU +EXE	[17]

EVI : éviscération NU: énucléation EXE : exentération

Des taux très variés ont été retrouvés au Nigéria avec un maximum de 5 % dans l’État d'Ebonyi où la population considérée est essentiellement rurale et sujette à des retards de prise en charge plus conséquents [18].

Le sex-ratio était estimé à 1,38 dans notre étude, correspondant aux données de la littérature où toutes les études retrouvaient une prédominance masculine [2, 25].

La prédominance masculine peut s'expliquer par une plus forte exposition des hommes aux traumatismes et leur acceptation de ce type de chirurgie qui est plus grande par rapport aux femmes qui préfèrent des mesures plus conservatrices [25].

L’âge moyen des patients éviscérés dans notre série était de 58 ans, ce qui est supérieur aux données de la littérature à savoir 37 ans en Iran [13], 40 ans au Togo [25], 43 ans au Népal [19], 50 ans en Arabie Saoudite [1] et 51 ans en Inde [6], ceci étant très probablement dû au fait que les enfants de moins de 16 ans sont pris en charge au niveau des hôpitaux pédiatriques. Ces hôpitaux-là n'ont pas été inclus dans notre échantillonnage.

La principale indication retrouvée était traumatique à 39 %, similaire aux résultats retrouvés en Iran [13] et en Arabie Saoudite [1]. Au Togo, la principale indication était le staphylome cornéen qu'il soit d'origine infectieuse ou suite à une brûlure cornéenne [25]. Au Nigéria (Sokoto), la principale indication des éviscérations reste les étiologies infectieuses alors que l’énucléation et l'exentération sont principalement réalisées pour des causes tumorales, essentiellement chez les enfants. Au Ghana, Cameroun, Népal et Nigéria (Ebonyi), les principales indications étaient infectieuses, englobant les endophtalmies et les panophtalmies [11, 15, 17, 18, 19] (Tableau VI).

**Tableau VI:** Indications des chirurgies mutilantes au sein de différents services d'ophtalmologie Indications for mutilating surgeries in different ophthalmology departments

Pays		Principales indications	Type de chirurgie	Références
Togo		Staphylome cornéen 37 %	EVI +ENU +EXE	[25]
Ghana		Infections 47,9 %	EVI +ENU	[15]
Cameroun		Panophtalmie/endophtalmie 47,9 %	EVI +ENU +EXE	[11]
Népal		Panophtalmie 31,71 %	EVI +ENU	[19]
Nigeria	Ebonyi	Panophtalmie 60,6 %	EVI +ENU +EXE	[18]
	Sokoto	Tumeurs 41,4 %	EVI +ENU +EXE	[17]
Iran		Traumatismes 33,2 %	ENU	[13]
Arabie Saoudite		Traumatismes 34,5 %	EVI +ENU	[1]

EVI : éviscération NU: énucléation EXE : exentération

Bien que les antécédents traumatiques soient retrouvés chez 41 % de nos patients, ces derniers ont conduit à indiquer une éviscération dans 39 % des cas seulement, pour les 2 % des cas restants l’éviscération était indiquée suite à une endophtalmie post traumatique.

Dans notre étude, nous avons retrouvé un seul cas où l’éviscération a été faite « à chaud » dans les suites immédiates d'un traumatisme balistique ayant induit une éviscération presque totale du globe.

Nous avons retrouvé 16 patients, tous âgés de moins de 40 ans, qui ne se plaignaient pas de douleur et où l'indication chirurgicale était purement esthétique sur un œil perdu.

Le type d'anesthésie utilisé dans notre série était une anesthésie générale dans 56 %. La majorité des études rapporte l'usage d'une anesthésie générale essentiellement dans les séries comportant un effectif considérable d'enfants. Comme cette série pakistanaise rapportée par Baig *et al.* où l'anesthésie générale est pratiquée dans 87,9 % [2]. A l'inverse, une série togolaise rapportée par Vonor *et al.* mentionne l'usage de l'anesthésie péribulbaire dans 64 % [25].

Certaines études préconisent d'utiliser de la ropivacaïne 1 % (Naropeine) en rétrobulbaire et ce quel que soit le type d'anesthésie utilisé pour prévenir les douleurs post-opératoires retrouvées chez 65 % des patients de notre série [3, 4, 26]. En effet, cette molécule a une durée d'action prolongée supérieure à celle de l'association bupivacaïne 0,75 % et lidocaïne 2 %, fréquemment utilisée. Son utilisation en fin d'intervention offre un plus faible risque de douleur et d'hémorragie postopératoires et une moindre neurotoxicité et cardiotoxicité. Le pourcentage d'implantation dans notre série est de 57 %, ce qui est faible par rapport aux recommandations actuelles qui mettent l'accent sur le comblement quasi-systématique des cavités anophtalmes soit par des implants orbitaires, soit par des greffes dermo-graisseuses.

Dans notre étude, l'implantation intra-sclérale a été réalisée à base d'implants inertes non colonisables en silicone ou en PMMA. D'autres matériaux bio colonisables peuvent être utilisés lors de l'implantation, qui sont à base de biocéramiques macroporeuses en hydroxyapatite, en corail ou en alumine [20]. Avec un recul de 8 ans en moyenne, nous avons noté un taux de complications estimé à 20 %.

La principale complication retrouvée était l'extériorisation de l'implant intra-scléral dans 10 % des cas. Selon l’étude de Gupta et *al.,* cette complication serait influencée par le degré d'expérience voire de compétence du chirurgien [14].

**Figure 7:**
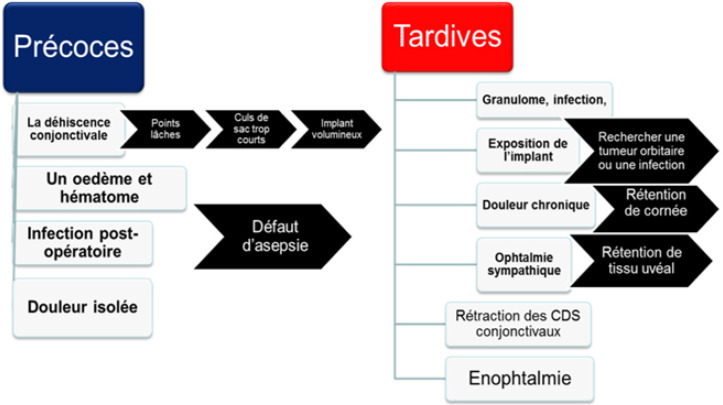
Les différentes complications décrites après une éviscération et leurs étiologies The different complications described after evisceration and their etiologies

Néanmoins les complications pouvant théoriquement survenir sont décrites figure 7 [24]. Le nombre d'ocularistes dans les régions concernées par notre étude était de 3 à Alger, 1 à Tizi Ouzou, 2 à Oran et 1 à Tlemcen. Ainsi, 48,5 % des patients ont été obligés de couvrir des distances supérieures à 100 kilomètres pour accéder à l'oculariste le plus proche. Ce nombre a augmenté ces dernières années avec l'ouverture de nouveaux laboratoires dans la région de Sétif, d'Alger et de Mascara respectivement à l'est, au centre et à l'ouest de l'Algérie.

En Algérie, la sécurité sociale ne prend en charge ni les honoraires de consultation de l'oculariste ni le coût de la prothèse, sachant que le coût moyen d'une prothèse varie entre 40 000 DA et 100 000 DA soit entre 200 et 500 €.

La plupart des ocularistes travaillent dans leur laboratoire personnel et le patient est libre de choisir son propre oculariste.

Le délai de prise en charge par l'oculariste dépend du temps de cicatrisation à savoir à partir de la 6^e^ semaine en post-opératoire.

Les suites défavorables d'un traumatisme oculaire ont été retrouvées au premier plan parmi les indications d’éviscération dans notre étude, soulevant ainsi l'intérêt des programmes nationaux visant à sensibiliser la population et axés sur la prévention primaire (contre les accidents domestiques, de la circulation, de la voie publique, etc.), et l'intérêt d'autres études prospectives qui les évalueront. La prévention des infections endoculaires retrouvées en second plan, essentiellement post-opératoires, est également une autre bataille qui doit être menée côte à côte par les ophtalmologistes et le personnel paramédical du bloc opératoire, car si dans 80 % des cas les germes responsables d'endophtalmie sont des germes de la flore conjonctivale du patient lui-même, la proportion d'infections nosocomiales reste non négligeable et peut être prévenue par une meilleure sensibilisation des praticiens de santé sur le nettoyage et l'asepsie de l'environnement et de l'instrumentation utilisée, le lavage chirurgical et l'antisepsie de l'opérateur, et par le nettoyage, l'antisepsie et l'antibioprophylaxie du patient [5].

Concernant les autres indications d’éviscération oculaire identifiées par notre étude, il s'agit de pathologies purement ophtalmologiques dont seule une prise en charge précoce et adéquate peut prévenir l'aggravation et donc le recours ultérieur à une chirurgie mutilante. Nous insistons ainsi sur une meilleure formation médicale continue des ophtalmologistes.

## Conclusion

Notre étude menée au nord de l'Algérie sur les éviscérations oculaires, nous a permis de déduire que cette chirurgie est rarement réalisée en ophtalmologie. Ses principales indications sont post-traumatiques et postinfectieuses. Perdre un œil est toujours vécu comme une tragédie et peut être dévastateur à tout âge, retentissant sur l'image et l'estime de soi. Le soutien psychologique est donc indispensable. La décision de réaliser une chirurgie mutilante doit être prise de manière collégiale après avoir épuisé toutes les autres alternatives thérapeutiques conservatrices [25]. La technique chirurgicale étant de réalisation facile, seules la minutie dans le geste, la rigueur et une meilleure formation des chirurgiens oculoplasticiens et ophtalmologistes sont les garants d'un faible taux de complications post-opératoires. Un suivi régulier des patients et une collaboration étroite avec les ocularistes sont des impératifs à respecter. La prévention des chirurgies mutilantes passe par le diagnostic précoce et le traitement adapté des pathologies ophtalmologiques et des traumatismes.

## Contribution des auteurs

AMMA Amine : conception de l’étude, développement et méthodologie, collecte des données, analyse/interprétation des données, rédaction du manuscrit, révision du manuscrit.

LAKHDAR FOUATIH Aïcha : développement et méthodologie, collecte des données, analyse/interprétation des données, révision du manuscrit.

HAMMAD Lamine : collecte des données, analyse/interprétation des données.

IDDER Aïcha : conception de l’étude, développement et méthodologie, collecte des données, analyse/interprétation des données, révision du manuscrit.

## Liens d'intérêts

Les auteurs déclarent qu'ils n'ont aucun lien d'intérêts.
